# Mid-life sleep is associated with cognitive performance later in life in aging American Indians: data from the Strong Heart Study

**DOI:** 10.3389/fnagi.2024.1346807

**Published:** 2024-06-05

**Authors:** Luciana Mascarenhas Fonseca, Myles G. Finlay, Naomi S. Chaytor, Natalie G. Morimoto, Dedra Buchwald, Hans P. A. Van Dongen, Stuart F. Quan, Astrid Suchy-Dicey

**Affiliations:** ^1^Elson S. Floyd College of Medicine, Washington State University, Spokane, WA, United States; ^2^Programa Terceira Idade (PROTER, Old Age Research Group), Department and Institute of Psychiatry, University of São Paulo School of Medicine, São Paulo, Brazil; ^3^Institute for Research and Education to Advance Community Health, Elson S. Floyd College of Medicine, Washington State University, Pullman, WA, United States; ^4^Sleep and Performance Research Center, Washington State University, Spokane, WA, United States; ^5^Division of Sleep and Circadian Disorders, Departments of Medicine and Neurology, Brigham and Women’s Hospital, Harvard Medical School, Boston, MA, United States; ^6^Division of Sleep Medicine, Harvard Medical School, Boston, MA, United States; ^7^Division of Pulmonary, Allergy, Critical Care and Sleep Medicine, University of Arizona College of Medicine, Tucson, AZ, United States

**Keywords:** Native Americans, health disparities, sleep quality, cognitive impairment, polysomnography

## Abstract

**Background:**

Sleep-related disorders have been associated with cognitive decline and neurodegeneration. American Indians are at increased risk for dementia. Here, we aim to characterize, for the first time, the associations between sleep characteristics and subsequent cognitive performance in a sample of aging American Indians.

**Methods:**

We performed analyses on data collected in two ancillary studies from the Strong Heart Study, which occurred approximately 10 years apart with an overlapping sample of 160 American Indians (mean age at follow-up 73.1, standard deviation 5.6; 69.3% female and 80% with high school completion). Sleep measures were derived by polysomnography and self-reported questionnaires, including sleep timing and duration, sleep latency, sleep stages, indices of sleep-disordered breathing, and self-report assessments of poor sleep and daytime sleepiness. Cognitive assessment included measures of general cognition, processing speed, episodic verbal learning, short and long-delay recall, recognition, and phonemic fluency. We performed correlation analyses between sleep and cognitive measures. For correlated variables, we conducted separate linear regressions. We analyzed the degree to which cognitive impairment, defined as more than 1.5 standard deviations below the average Modified Mini Mental State Test score, is predicted by sleep characteristics. All regression analyses were adjusted for age, sex, years of education, body mass index, study site, depressive symptoms score, difference in age from baseline to follow-up, alcohol use, and presence of *APOE e4* allele.

**Results:**

We found that objective sleep characteristics measured by polysomnography, but not subjective sleep characteristics, were associated with cognitive performance approximately 10 years later. Longer sleep latency was associated with worse phonemic fluency (*β* = −0.069, *p* = 0.019) and increased likelihood of being classified in the cognitive impairment group later in life (odds ratio 1.037, *p* = 0.004). Longer duration with oxygen saturation < 90% was associated with better immediate verbal memory, and higher oxygen saturation with worse total learning, short and long-delay recall, and processing speed.

**Conclusion:**

In a sample of American Indians, sleep characteristics in midlife were correlated with cognitive performance a decade later. Sleep disorders may be modifiable risk factors for cognitive impairment and dementia later in life, and suitable candidates for interventions aimed at preventing neurodegenerative disease development and progression.

## Introduction

Sleep quality is a complex construct that can be measured both objectively and subjectively, and includes quantitative aspects of sleep, such as duration and latency, but also qualitative aspects, such as how restored someone feels after sleeping (Buysse et al., 1989; Nelson et al., 2022). Sleep–wake cycle disruptions are frequent during the course of dementia and the prodromic phases of neurodegeneration (Lyketsos et al., 2002; Moran et al., 2005; Zhao et al., 2016). The relationship between sleep and dementia is bidirectional; while physiological and neuropathological aspects of dementia reduce the quality of sleep, poor sleep quality may also increase the risk of neurodegenerative diseases and dementia (McCoy and Strecker, 2011; Lim et al., 2014; Borges et al., 2019). Sleep and nighttime behavior disturbances are among the most important symptoms of dementia, leading to a need for long-term care, institutionalization, and caregiver burden in various populations (Hope et al., 1998; Moran et al., 2005; Fonseca et al., 2021), with attendant costs to individuals and society.

Poor sleep quality and short sleep duration during midlife have been associated with increased amyloid-beta deposition and the acceleration of regional brain hypometabolism (Choe et al., 2019), respectively. A study in young adults also showed that certain sleep disturbance patterns (such as elevated slow waves and daytime sleepiness) were associated with genetic liability for Alzheimer’s Disease (AD) before the appearance of other AD biomarkers (Muto et al., 2020). Disturbed sleep may also impact negatively on sleep-dependent consolidation of memory and on cerebral spinal fluid mediated clearance of metabolic residues from the brain, which are linked processes (Fultz et al., 2019) also known to be correlated with amyloidosis and AD (Bateman et al., 2007).

Sleep plays an important role in cognitive performance (Walker, 2009; Tsapanou et al., 2020), and the importance of sleep for the codification and consolidation of memory as well as for the retrieval of new declarative memories (Walker, 2009) is well established. Without adequate sleep, hippocampal function is disrupted and, consequently, so is the ability to record new experiences (Eichenbaum, 2004). Good sleep quality has been shown to correlate with better cognitive function in children (El-Sheikh et al., 2019) and has an impact on cognitive function during the waking period in adolescence, contributing to learning (Tarokh et al., 2016). In 141 middle-aged participants on the Sleep Heart Health Study, more severe oxygen saturation was associated with worse motor speed and processing speed performance after 9–40 months (Quan et al., 2006). A 25-year follow-up study including 7,959 participants demonstrated that persistent short sleep duration (<6 h) at ages 50, 60 and 70 was associated with a 30% increase in the risk of dementia compared with 6–8 h of sleep duration (Sabia et al., 2021). Correlations between sleep disorders and cognitive measures in healthy individuals (Walker, 2009; Tsapanou et al., 2016) and those with AD (Bonanni et al., 2005; Shin et al., 2014) have been found, with poorer nighttime sleep quality being associated with worse performance on praxis, immediate recall, and global cognition in AD patients (Shin et al., 2014). Furthermore, daytime sleepiness has been associated with cognitive performance deficits in both cross-sectional and longitudinal studies (Foley et al., 2001; Ohayon and Vecchierini, 2002; Bonanni et al., 2005; Merlino et al., 2010; Elwood et al., 2011; Tsapanou et al., 2016; Muto et al., 2020), as well as with incident dementia in many longitudinal studies (Foley et al., 2001; Elwood et al., 2011; Schlosser Covell et al., 2012). Some studies have also found sleep-disordered breathing to be associated with generally impaired cognitive function (Cohen-Zion et al., 2001; Spira et al., 2008), particularly impaired executive function (Ju et al., 2012) and memory (Aloia et al., 2003; Ju et al., 2012).

There is a growing body of evidence showing that there are racial/ethnic disparities related to sleep quality (Kalliny and McKenzie, 2017; Pandi-Perumal et al., 2017; Prasad et al., 2018; Denney et al., 2023). Sleep quality is influenced by various social, cultural, and genetic factors (Kalliny and McKenzie, 2017; Prasad et al., 2018). It is believed that the racial differences found in sleep disorders are due to several environmental stressors in personal health, such as socioeconomic inequalities, acculturation, and racism (Ruiter et al., 2011; Egan et al., 2017; Prasad et al., 2018; Liu et al., 2021). Sleep-related disorders, such as obstructive sleep apnea, and excessive daytime sleepiness have been linked to poor health outcomes, including cardiovascular disease (Ruiter et al., 2010; Drager et al., 2017; Egan et al., 2017). Also, there is evidence that both sleep disorders (Ruiter et al., 2010; Chen et al., 2015) and cardiovascular diseases (Egan et al., 2017; Zuma et al., 2021) are influenced by race/ethnicity. Furthermore, sleep-related disorders as well as vascular factors that can be associated with sleep deficiencies, such as arterial hypertension, diabetes, and hypercholesterolemia, are known risk factors for cognitive impairment and AD and related dementias. American Indians have a high prevalence of cerebral vascular disease (Hutchinson and Shin, 2014; Suchy-Dicey et al., 2017), stroke (Harris et al., 2015) and type 2 diabetes (Centers for Disease Control and Prevention, 2003; Cholerton et al., 2019), along with the presence of other health disparities (Hutchinson and Shin, 2014), but the impact of sleep-related disorders and their relationships with dementia are understudied in this population.

A recent study of self-reported sleep duration and mortality risk in different racial and ethnic groups found elevated risks of all-cause mortality for those who reported sleeping less than 5 h or more than 9 h per day, with variation by racial and ethnic identification (Denney et al., 2023). This study found that relative to White individuals, a significantly higher proportion of American Indians reported sleeping less than 5 h or more than 9 h. However, the authors acknowledged that the study was underpowered to estimate the association between sleep duration and mortality for this specific population.

To our knowledge, there are no studies investigating sleep-related disorders and their cognitive consequences focused specifically on American Indians. Data are needed in this population to identify possible opportunities for primary and secondary prevention of cognitive decline. The aim of this study is to characterize, for the first-time, sleep characteristics and their relationships with cognitive performance and classification of cognitive impairment approximately 10 years later in a sample of American Indians. Based on data showing that prevalent health and racial/ethnic disparities may contribute to sleep disorders (Kalliny and McKenzie, 2017; Pandi-Perumal et al., 2017; Prasad et al., 2018), that sleep disorders are frequently linked to cognitive decline (Foley et al., 2001; Merlino et al., 2010; Tsapanou et al., 2016), and that older American Indians are at greater risk for cognitive decline and dementia (Centers for Disease Control and Prevention, 2003; Hutchinson and Shin, 2014; Suchy-Dicey et al., 2017), we hypothesize that poor sleep quality will be associated with worse cognitive performance 10 years later.

## Materials and methods

### Study setting

#### Study design and ethics

The current study involves secondary analyses of data collected in two multisite ancillary studies from the Strong Heart Study: the Sleep Heart Health Study (Quan et al., 1997) (SHHS), which includes polysomnography and self-reported sleep quality data, and the Cerebrovascular Disease and its Consequences in American Indians (CDCAI) study (Suchy-Dicey et al., 2016), which includes neuropsychological assessments. Written informed consent was obtained from all participants for both studies. Study procedures were approved by the appropriate Institutional Research Boards, Tribal Research Review Boards, or Tribal Councils.

#### Sleep Heart Health Study and Cerebrovascular Disease and its consequences in American Indians Study

Sleep data were collected in the SHHS, which enrolled participants from several observational studies of cardiovascular and respiratory disease including the Strong Heart Study (SHS). The SHHS took place from 1995 until 2009; its design and methods have been fully described elsewhere (Quan et al., 1997). Sleep data were collected during a home visit. This included a polysomnogram, measures of sleep-disordered breathing, and self-report assessments of poor sleep quality, snoring, insomnia, and daytime sleepiness from the SHHS Sleep Habits Questionnaire (Quan et al., 1997).

**Figure 1:**
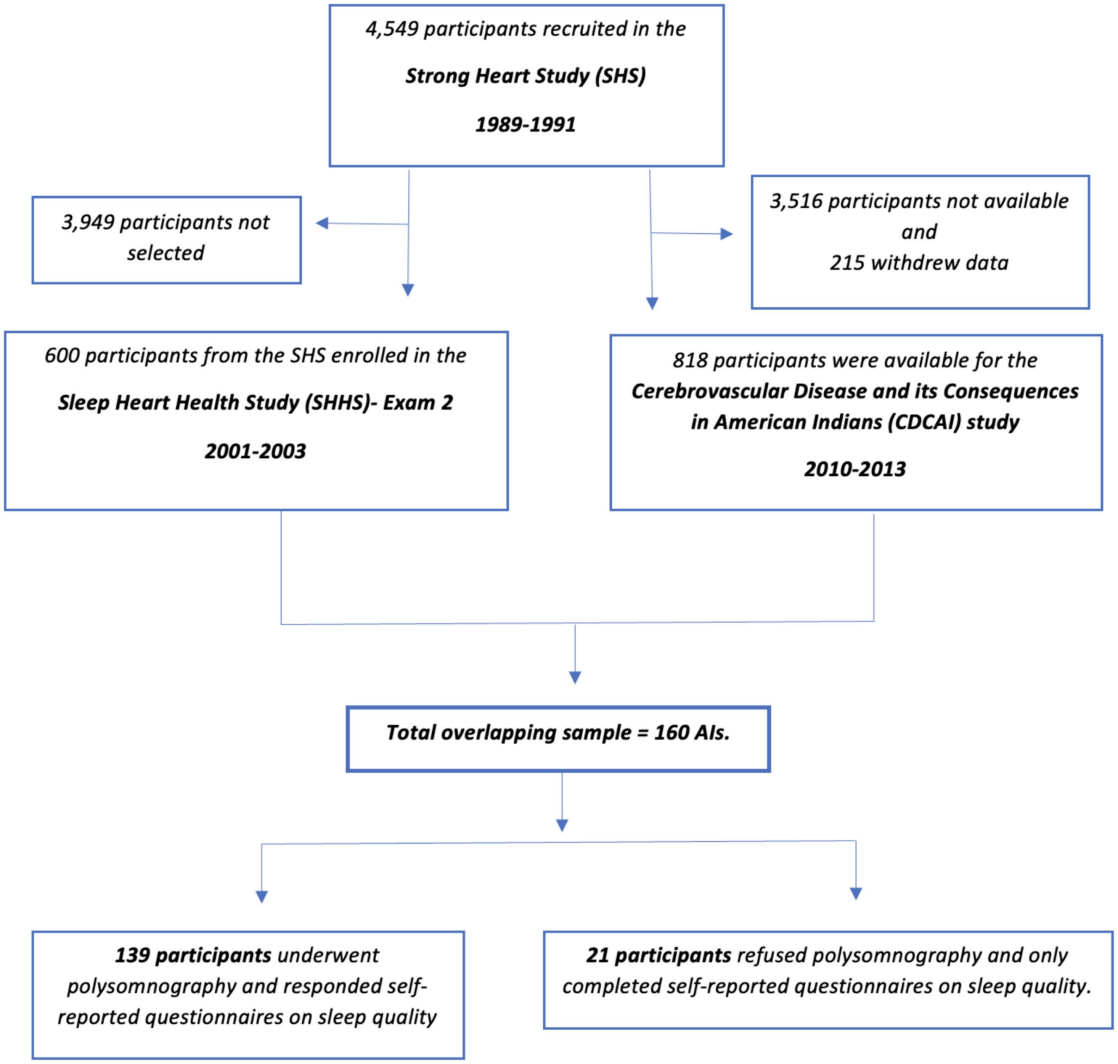
Flow diagram of participant inclusion in the study.

The CDCAI study is the largest cohort of aging American Indians that included neuropsychological data collection. The study took place from 2010 until 2013 and included 818 participants from the SHS recruited from three study sites – the Northwest Plains, Southern Plains, and Southwest region – representing 11 of the 13 original SHS communities. The design and methods of the CDCAI study have been fully described elsewhere (Suchy-Dicey et al., 2016). See Figure 1 for a diagram of all three studies involved.

### Study sample

Of the 818 American Indians who completed neuropsychological testing in the CDCAI study (mean age of 73.0 years during the years 2010–2013, standard deviation 5.9 years; 555 females, 67.9%), we identified 160 who also had sleep data from the SHHS (mean age of 62.6 years during the years 1995–1998, standard deviation 5.4 years; 111 females, 69.3%). The participant inclusion diagram is shown in Figure 1.

### Data collection

#### Sleep measures

Sleep characteristics assessed in the SHHS were identified by (1) polysomnography using the validated portable PS-2 polysomnography system (Compumedics, Abottsville, AU) (Redline et al., 1998), including electroencephalogram (derivations C3/A1 and C4/A2), left and right electrooculogram, submental chin electromyogram, and electrocardiogram (modified Lead I), as well as measurements of abdominal and thoracic excursions, oxyhemoglobin saturation, airflow, body position, and ambient light; (2) sleep-disordered breathing evaluated using apnea-hypopnea and respiratory disturbance indices; and (3) self-reported indicators of poor sleep quality (delayed sleep onset, short sleep duration, snoring, and insomnia) and daytime sleepiness collected with the SHHS Sleep Habits Questionnaire (Quan et al., 1997). Polysomnography data were available for 139 participants, the remaining participants (*n* = 21) declined to undergo polysomnography. Scoring of sleep architecture was performed using Rechtschaffen and Kales criteria (Rechtschaffen and Kales, 1968).

Sleep variables used for this study were: *s*leep onset time, total sleep time, sleep efficiency, sleep latency, rapid eye movement (REM) sleep latency, percentage sleep time in REM sleep, percentage sleep time in stage 1 sleep, percentage sleep time in stage 2 sleep, percentage sleep time in slow wave sleep (stages 3 and 4 were combined in slow wave sleep), obstructive apnea hypopnea index (irrespective of any associated oxygen desaturation as well as associated with oxygen desaturation ≥4%), arousal index, average oxygen saturation, lowest oxygen saturation during non-REM (NREM) sleep, lowest oxygen saturation during REM sleep, number of oxygen desaturations, respiratory disturbance index (RDI) (associated oxygen desaturation ≥3%), central apnea index, lowest overall oxygen saturation, oxygen saturation classification (defined as: normal or minimal >90%, mild desaturation 80–90%, and severe desaturation <80%), and percent of sleep time with oxygen saturation < 90%. Self-reported delayed sleep onset, as well as Epworth Sleepiness Scale (ESS) score and daytime sleepiness classification (ESS > 10) were also included. Detailed information and metrics have been fully described elsewhere (Quan et al., 1997, 2007).

#### Neuropsychological assessment

The neuropsychological assessment in the CDCAI study included (1) the Modified Mini Mental State Test (3MSE) (Teng and Chui, 1987), a measure of general cognition based on the Mini Mental State Test (Folstein et al., 1975); (2) the Wechsler Adult Intelligence Scale (WAIS-IV) digit symbol coding test (Wechsler, 2008), a measure of processing speed; (3) the California Verbal Learning Test, Second Edition, Short Form (CVLT-SF) (Delis et al., 2000), which measures episodic verbal learning (total learning), recall (short-delay and long-delay free recall), and recognition discriminability; and (4) the Controlled Oral Word Association (COWA) test (Ruff et al., 1996), which provides a measure of verbal phonemic fluency. Measurements were grouped in cognitive domains as follows: (1) *Memory*, CVLT-SF subtests for total learning, short-delay free recall, long-delay free recall, and recognition discriminability; (2) *Processing speed,* WAIS-IV digit symbol coding test (number correctly coded in 120 s); (3) *Executive Function*, COWA test; and (4) *Global cognition*, 3MSE test. All cognitive variables are continuous raw scores, with higher scores corresponding to better performance.

Subjects were categorized as having cognitive impairment if their individual z-scores for the 3MSE test, based on the mean and standard deviation of the sample during the 10-year follow-up (*N* = 160), were less than −1.5 (i.e., more than 1.5 standard deviations below the group average 3MSE score). While there is no established cutoff point for this population, this classification considers the individual in comparison with the group of the same race/ethnicity, and a criterion of 1.5 standard deviations from the mean is a common approach to define mild cognitive impairment (Petersen et al., 2001, 2014).

#### Other instruments and measures

During the CDCAI study, participants completed self-administered questionnaires for sociodemographic data, including sex (self-reported as male or female), education (years), self-reported alcohol consumption (current or former drinker), and height and weight from which body mass index (BMI) was calculated. The Centers for Epidemiologic Studies – Depression (CESD) scale assessed symptoms of depression (range 0–60, higher scores indicate greater depressive symptoms) (Radloff, 1977). To account for the time interval between the studies, we also included a variable for participants’ age difference from baseline to follow-up (age at CDCAI – age at SHHS). Blood was collected for genetic assessment of the presence of the *apolipoprotein E e4* (*APOE e4*) allele, a risk factor for dementia (Gharbi-Meliani et al., 2021), determined with isoelectric focusing.

### Statistical analysis

Descriptive statistics were calculated and presented as absolute and relative frequencies or as means and standard deviations for the sample. The Kolmogorov–Smirnov test was used to test for normality, and nonparametric analysis was used when needed. Relationships among sleep variables were assessed by means of correlations.

To determine whether sleep characteristics were associated with cognitive performance and cognitive impairment assessed approximately 10 years later, we first calculated correlations between each of the sleep measures from the SHHS and each of the cognitive outcomes from the CDCAI study using Spearman rho as data did not follow a normal distribution. For variables that were significantly correlated, linear regression analyses were conducted with each cognitive outcome as the dependent variable and each sleep measures as the independent variable, adjusting for age, sex, years of education, BMI, study site, CESD depressive symptoms score, participants’ age difference from baseline to follow-up, alcohol use, and *APOE e4* genotype.

The degree to which cognitive impairment was predicted by sleep characteristics was examined with odds ratios determined with logistic regression, adjusting for age, sex, years of education, BMI, site, CESD depressive symptoms score, participant’s age difference from baseline to follow up, alcohol use, and *APOE e4* genotype. Sleep variables were included in this logistic regression model if: (1) there was an association of the variable with cognitive outcomes in the bivariate analyses; (2) any collinearity was resolved; and (3) there was an empirical or theoretical rationale for including the variable in the model. To reduce the dimensionality of the comparisons, this analysis was performed as a stepwise regression analysis.

Examination of correlations between predictor variables revealed pairs of highly correlated sleep variables. We found collinearity for oxygen desaturation index, respiratory disturbance index, obstructive apnea hypopnea index, lowest overall oxygen saturation level, and lowest % of oxygen saturation during REM and NREM sleep. When collinearity was encountered, we selected only those variables that were correlated with cognitive outcomes in the bivariate analysis. Any remaining collinearity was resolved by choosing the variables with the greatest clinical utility (e.g., percent of sleep time with oxygen saturation < 90% instead of average oxygen saturation).

The type I error threshold for statistical significance was set at 0.05. This study involves a discovery-based approach in an exploratory analysis of sleep and cognitive data reported for the first time for American Indians. Therefore, to control type I error given multiple comparisons, but still be able to identify as many significant associations as possible, we used false discovery rate corrections to *p*-values, which is a less conservative approach than Bonferroni corrections. Statistical analyses were carried out using the Statistical Package for the Social Sciences (SPSS), version 28 for Windows.

## Results

### Demographic characteristics

Table 1 shows the demographic characteristics of the participants as well as results from the neuropsychological assessment battery and CESD depression scale. Demographic data of this subgroup are comparable to data from the entire CDCAI cohort, as are the results of the cognitive tests. An exception was the mean 3MSE, a test of general cognition with a greater range of scores (1–100). The mean 3MSE score for this group (mean = 93.9/ standard deviation = 11.8) was higher than the mean for the entire CDCAI cohort (mean = 88.5/ standard deviation = 10.1). Of the 160 participants of this group, 111 (69.4%) were female, with a mean age of 62.6 years at the first timepoint (SHHS) and 73.1 years at the second timepoint (CDCAI). In the sample, 50% of the participants were from the Northern Plains, 37.5% were from the Southern Plains, and 12.5% were from the Southwest region. The mean interval between studies measured by participants’ age difference from baseline to follow-up was 10.4 years, ranging from 7 to 16 years. Participants had from 6 to 20 years of education, with 80% of the sample having completed high school or more. Twenty three percent of the sample were carriers of the *APOE e4* allele.

**Table 1:** Demographic characteristics and neuropsychological assessment.

Total sample: *N* = 160	SHHS (2001–2003)	CDCAI Study (2010–2013)
Age, mean (SD) / range	62.66 (5.41) / 55–78	73.11 (5.69) / 65–90
Self-reported sex, *n* female (%)	111 (69.37%)	
Study site, *n* (%)		
Northern Plains	80 (50.0%)	
Southern Plains	60 (37.5%)	
Southwest	20 (12.5%)	
Difference in age (from baseline to follow-up), mean (SD) / range		10.44 (1.34)/ 7–16
Education (years), mean (SD) / range		12.85 (2.52) / 6–20
Education, *n* (%)		
Up to 11th grade		32 (20.00%)
High school or GED		49 (30.62%)
Any college		59 (36.87%)
Bachelor’s degree or more		20 (12.50%)
*APOE e4* allele carrier, *n* (%)		37 (23.12%)
Body Mass Index (*N =* 156), mean (SD) / range	32.01 (5.78) / 18.29–49.77	31.65 (5.98) / 18.13–46.56
Depression (CESD score; *N =* 156), mean (SD) / range		10.49 (7.93) / 0–41
Alcohol use, n (%), ever (yes)		118 (73.8%)
Cognitive test scores, mean (SD) / range		
CVLT-SF (*N* = 152)		
Total learning		22.89 (5.05) / 8–35
Short-delay free recall		5.97 (2.05) / 0–9
Long-delay free recall		5.27 (2.29) / 0–9
Recognition discriminability		2.59 (0.76) / 0.30–3.50
COWA (*N =* 156)		23.98 (12.06) / 0–58
WAIS-IV-digit symbol coding test (*N* = 155)		43.97 (15.98) / 0–91
3MSE (*N =* 156)		93.99 (11.80) / 18–100

SHHS, Sleep Heart Health Study; CDCAI, Cerebrovascular Disease and its Consequences in American Indians; SD, standard deviation; GED, General education development test; CESD, Center for Epidemiological Studies – Depression scale; CVLT-SF, California Verbal Learning Test, Second Edition, Short Form; COWA, Controlled Oral Word Association test; WAIS-IV, Wechsler Adult Intelligence Scale, Fourth Edition; 3MSE, Modified Mini Mental State Test.

### Sleep characteristics

Table 2 presents the sleep variables. Mean sleep duration was 371.3 min (6.19 h), with a standard deviation of 73.5 min. Of the total sample, 18% slept less than 5 h, 41.7% slept for less than 6 h, and 74.8% slept for less than 7 h, as measured by polysomnography. 33 participants had a sleep latency of more than 30 min (23.7% of 139). Forty percent of the sample had moderate to severe respiratory disturbance as determined by a respiratory disturbance index ≥15 events per hour. Moreover, 55.6% were classified with mild oxygen desaturation (80–90% saturation) and 24.3% were classified with severe oxygen desaturation, as measured during polysomnography.

**Table 2:** SHHS sleep characteristics.

Polysomnography	
Sleep onset time (clock time; *N* = 139), mean (SD in minutes) / range	10:22 pm (59.16) / 23–291
Total sleep time (minutes; *N* = 139), mean (SD) / range	371.32 (73.52) / 217–547
Sleep efficiency (%; *N* = 139), mean (SD) / range	77.41 (12.80) / 39–96
Sleep latency (minutes; *N* = 139), mean (SD) / range	22.63 (31.42) / 0–180
REM latency (minutes from sleep onset; *N* = 137), mean (SD) / range	105.50 (64.97) / 6–405
Percentage sleep time in REM sleep (*N* = 137), mean (SD) / range	20.86 (6.00) / 1–39
Percentage sleep time in stage 1 sleep (*N* = 139), mean (SD) / range	6.15 (3.74) / 0–23
Percentage sleep time in stage 2 sleep (*N* = 138), mean (SD) / range	58.33 (10.43) / 32–87
Percentage sleep time in slow wave sleep (*N* = 139), mean (SD)/ range	14.65 (10.91) / 0–39
Obstructive Apnea Hypopnea Index (≥4% desaturation, events per hour, *N* = 138), mean (SD) / range	2.70 (7.62)/ 0–66
Obstructive Apnea Hypopnea Index (all desaturations, events per hour; *N* = 138), mean (SD) / range	3.12 (8.19) / 0–70
Arousal index (events per hour; *N* = 137), mean (SD) / range	17.53 (9.95) / 4–78
Average oxygen saturation (%; *N* = 138)^2^, mean (SD) / range	93.76 (1.81) / 88–98
Lowest % of oxygen saturation during NREM sleep (*N* = 139), mean (SD) / range	85.52 (5.82) / 54–94
Lowest % of oxygen saturation during REM sleep (*N* = 137), mean (SD) / range	82.91 (6.93) / 60–95
Number of desaturations (≥ 3%; *N* = 138), mean (SD) / range	109.28 (84.34) / 4–408
Respiratory Disturbance Index (3% desaturation, events per hour; *N* = 138)ƚ^2^, mean (SD) / range	17.82 (17.25) / 0–93
Respiratory Disturbance Index classification (*N* = 138), *n* (%)	
Normal (0–4 events/h)	30 (18.75%)
Mild (5–14 events/h)	44 (27.50%)
Moderate (15–29 events/h)	39 (24.37%)
Severe (≥30 events/h)	25 (15.62%)
Respiratory Disturbance Index ≥15 events/h (moderate and severe; *N* = 139)^2^, *n* (%)	64 (40.00%)
Central Apnea Index (all desaturations, events per hour; *N* = 138)^2^, mean (SD) / range	0.38 (1.50) / 0–14
Minimal oxygen saturation (%, *N* = 138), mean (SD) / range	82.09 (7.07) / 54–94
Oxygen saturation classification (*N* = 138), *n* (%)	
Normal or Minimal (>90%)	10 (6.25%)
Mild desaturation (80–90%)	89 (55.62%)
Severe desaturation (<80%)	39 (24.37%)
Percent of sleep time with oxygen saturation < 90% (*N* = 138), mean (SD) / range	5.41 (10.28) / 0–53
Self-reported sleep characteristics (Sleep Habits Questionnaire)	
Daytime sleepiness (*N* = 160), *n* (%)	
Never	38 (23.75%)
Rarely (Once/month or less)	45 (28.12%)
Sometimes (2–4x/month)	57 (35.62%)
Often (5–15x/month)	10 (6.25%)
Almost always (16–30x/month)	10 (6.25%)
Epworth Sleepiness Scale (ESS) score (*N* = 160), mean (SD)/ range	6.20 (4.05) / 0–21
Excessive daytime sleepiness (ESS > 10; *N* = 160), *n* (%)	32 (20.0%)
Delayed sleep onset (*N* = 160), *n* (%)	
Never	52 (32.50%)
Rarely (Once/month or less)	42 (26.25%)
Sometimes (2–4x/month)	51 (31.87%)
Often (5–15x/month)	11 (6.87%)
Almost always (16–30x/month)	4 (2.50%)

SHHS, Sleep Heart Health Study; NREM, non-REM sleep (stages 2, 3, and 4); SD, standard deviation.

Based on self-reported information on the Epworth Sleepiness Scale, 20% of the sample was classified as having excessive daytime sleepiness, with 48.1% out of the 160 participants reporting daytime sleepiness at least twice a month or more, and 6.2% reporting daytime sleepiness almost every day. Furthermore, out of the 160 study participants, 41.2% reported having delayed sleep onset at least twice a month, while 2.5% reported delayed sleep onset almost every day.

### Unadjusted correlations between sleep and cognitive variables

Table 3 shows the correlations between sleep characteristics and cognitive performance evaluated approximately 10 years later, adjusted for multiple comparisons using false discovery rate.

**Table 3:** Correlations between sleep characteristics and cognitive performance.

	Cognitive performance						
	Memory				Executive function	Processing speed	Global cognition
	CVLT-SF				COWA	WAIS-IV digit symbol coding test	3MSE
	Total learning	Short-delay free recall	Long-delay free recall	Recognition discriminability			
**Sleep characteristics**	*Spearman’s rho*						
Sleep onset time (*N* = 139)	0.051	−0.016	−0.021	0.046	−0.016	−0.050	0.014
Sleep duration (*N* = 139)	0.075	0.030	0.049	0.028	0.195*	0.077	0.100
Sleep efficiency (*N* = 139)	0.187*	0.116	0.121	0.059	0.172*	0.092	0.211*
Sleep latency (*N* = 139)	−0.063	−0.032	−0.055	−0.010	−0.190*	−0.168	−0.160
REM latency (*N* = 137)	−0.051	0.006	0.002	−0.003	−0.057	0.101	0.049
Percentage of sleep time in REM sleep (*N* = 137)	0.215*	0.095	0.170	0.136	0.136	0.073	0.046
Percentage of sleep time in stage 1 (*N* = 139)	−0.200*	−0.080	−0.062	−0.132	−0.127	−0.203*	−0.165
Percentage of sleep time in stage 2 (*N* = 138)	−0.027	−0.064	−0.013	−0.052	−0.032	−0.071	−0.006
Percentage of sleep time in slow wave sleep (*N* = 139)	0.023	0.021	0.004	0.063	0.005	0.123	0.032
Obstructive Apnea Hypopnea Index (all desats; *N* = 138)	0.080	0.010	0.091	0.020	−0.095	0.048	0.058
Obstructive Apnea Hypopnea Index (≥4% desats; *N* = 138)	0.144	0.088	0.119	0.086	−0.011	0.011	0.033
Arousal index (*N* = 137)	0.048	0.044	−0.001	0.018	0.045	0.029	0.026
Average oxygen saturation (*N* = 138)	−0.185*	−0.368**	−0.265**	−0.164	−0.067	−0.215*	−0.178*
Lowest % of oxygen saturation during NREM sleep (*N* = 139)	−0.089	−0.104	−0.103	0.020	−0.010	−0.060	−0.050
Lowest % of oxygen saturation during REM sleep (*N* = 137)	−0.192*	−0.150	−0.190*	−0.148	0.024	−0.160	−0.130
Number of desaturations (≥ 3%; *N* = 138)	0.167	0.025	0.096	0.102	−0.031	0.046	0.028
Respiratory Disturbance Index (3% desats; *N* = 138)	0.186*	0.109	0.129	0.090	0.007	0.020	0.048
Central Apnea Index (all desats; *N* = 138)	0.080	−0.027	0.033	−0.035	0.012	0.021	0.075
Minimal oxygen saturation (*N* = 138)	−0.130	−0.124	−0.151	−0.075	0.025	−0.093	−0.080
Percent of sleep time with oxygen saturation < 90% (*N* = 138)	0.166	0.229**	0.221*	0.131	−0.001	0.167	0.135
Epworth Sleepiness Scale (*N* = 160)	0.127	0.091	0.076	0.095	0.073	0.031	0.087
Excessive daytime sleepiness (*N* = 160)	0.074	0.034	0.073	−0.061	−0.031	−0.033	0.009
Delayed sleep onset (*N* = 160)	−0.045	−0.049	−0.045	−0.011	−0.071	−0.019	−0.095

SD, standard deviation; NREM, non-REM sleep (stages 2, 3, and 4); CVLT-SF, California Verbal Learning Test, Second Edition, Short Form; COWA, Controlled Oral Word Association test; WAIS-IV, Wechsler Adult Intelligence Scale, Fourth Edition; 3MSE, Modified Mini Mental State Test. *p < 0.05, **p < 0.01 (two-tailed) after false discovery rate correction.

#### Sleep and memory

Verbal list learning performance was positively correlated with sleep efficiency and respiratory disturbance index, and negatively correlated with percentage of sleep time in stage 1, average oxygen saturation, and lowest percent of oxygen saturation during REM sleep. Short-delay free recall was positively correlated with percent of sleep time with oxygen saturation below 90% and negatively correlated with average oxygen saturation (i.e., better oxygen saturation predicted impaired short-delay free recall). Long-delay free recall was positively correlated with percent of sleep time in REM and percent of sleep time with oxygen saturation below 90%, and negatively correlated with average oxygen saturation and lowest % of oxygen saturation during REM sleep (i.e., better oxygen saturation overall and during REM sleep predicted impaired long-delay free recall).

#### Sleep and executive functions

Total COWA score was positively correlated with sleep duration and sleep efficiency, and negatively correlated with sleep latency.

#### Sleep and processing speed

WAIS-IV digit symbol coding test performance was negatively correlated with percent of sleep time in stage 1 and average oxygen saturation.

#### Sleep and global cognition

3MSE score was positively correlated with sleep efficiency and negatively correlated with average oxygen saturation. Recognition discriminability measured by the CVLT-SF was not correlated with any of the sleep variables.

### Pairwise linear regression analyses

For the sleep and cognition variable pairs that were significantly correlated, separate linear regression analyses were conducted using each cognitive outcome as the dependent variable and the associated sleep variable as the independent variable (predictor), adjusting for the covariates. Table 4 shows the variable pairs that were statistically significant in these regression analyses.

**Table 4:** Significant associations between sleep characteristics and cognitive performance in pairwise linear regression models adjusted for age, BMI, sex, study site, years of education, CESD depressive symptoms score, age difference from baseline to follow-up, alcohol use, and presence of *APOE e4* allele.

	Unstandardized coefficient	Standardized coefficient	95% Confidence interval for *B*		*p*-value
	*B*	*β*	Lower bound	Upper bound	
**Sleep and memory**					
Dependent variable: CVLT-SF total learning score					
*Predictors: Average oxygen saturation*	−0.541	−0.199	−1.003	−0.078	0.022
*Percentage of sleep time in REM sleep*	0.145	0.178	0.015	0.275	0.029
Dependent variable: CVLT-SF short-delay free recall score					
*Predictors: Average oxygen saturation*	−0.415	−0.364	−0.613	−0.217	<0.001
Dependent variable: CVLT-SF long-delay free recall score					
*Predictor: Average oxygen saturation*	−0.264	−0.204	−0.485	−0.043	0.020
**Sleep and executive function**					
Dependent variable: COWA score					
*Predictor: Sleep latency*	−0.073	−0.196	−0.128	−0.017	0.011
**Sleep and processing speed**					
Dependent variable: WAIS-IV digit symbol coding test score					
*Predictor: Average oxygen saturation*	−1.629	−0.184	−2.997	−0.260	0.020

BMI, body mass index (measured during CDCAI study); CESD, Centers for Epidemiologic Studies- Depression scale; CVLT-SF, California Verbal Learning Test, Second Edition, Short Form; COWA, Controlled Oral Word Association test; WAIS-IV, Wechsler Adult Intelligence Scale, Fourth Edition.

#### Sleep and memory

More time in REM sleep predicted better performance on CVLT-SF total learning. Higher oxygen saturation predicted worse performance on CVLT-SF total learning, short-delay free recall, and long-delay free recall.

#### Sleep and executive functions

Longer sleep latency predicted worse performance on the COWA test.

#### Sleep and processing speed

More time in REM sleep predicted worse performance on the WAIS-IV digit symbol coding test.

### Odds of clinically relevant cognitive impairment

Table 5 shows the results of stepwise logistic regression for clinically relevant cognitive impairment, defined as >1.5 standard deviations below the average 3MSE score, with multiple sleep variables as predictors and adjusted for the covariates. All sleep variables with any significant correlations with cognition (Table 3) entered the model (i.e., sleep latency, sleep duration, sleep efficiency, percentage of time in stage 1, % sleep in REM, lowest % of oxygen saturation during REM, respiratory index 3%, % sleep time with oxygen <90%), except for average oxygen saturation due to collinearity with percent sleep time with oxygen saturation < 90%. Following completion of the stepwise regression process, the logistic regression model was significant (*X*^2^_16_ = 56.20, *p* < 0.001), explaining 57.8% of the variance (Nagelkerke *R*^2^) and correctly classifying 88.5% of cases. Sleep latency was the only sleep variable that was retained through the stepwise regression process. Longer sleep latency (expressed in minutes) was associated with greater likelihood of being classified in the cognitive impairment group, with odds ratio 1.035 (95% confidence interval: 1.010–1.061; *p* = 0.005).

**Table 5:** Associations between sleep characteristics and the odds of cognitive impairment, adjusted for age, sex, BMI, site, years of education, CESD depressive symptoms score, age difference from baseline to follow-up, alcohol use, and presence of the APOE e4 allele (*N* = 130).

	Odds ratio	95% CI	*p*-value
Age (years)	1.189	1.033–1.368	0.016
Sex (female)	0.089	0.010–0.759	0.027
Education (years)	0.564	0.384–0.827	0.003
Site (Southern compared to Northern Plains)	19.334	2.378–157.181	0.006
Site (Southwest compared to Northern Plains)	37.615	2.165–653.632	0.013
Sleep latency (minutes)	1.035	1.010–1.061	0.005

The variables included in the model are sleep latency, sleep duration, sleep efficiency, percentage of sleep time in stage 1, % sleep in REM, lowest % of oxygen saturation during REM, respiratory index 3%, % sleep time with oxygen < 90%, as well as covariates for age, BMI, sex, site, years of education, CESD (Centers for Epidemiologic Studies- Depression) scale scores, age difference (from baseline to follow-up), alcohol use, and presence of the *APOE e4* allele.

Age, sex, years of education, and study site were significant as covariates. Older individuals (with age expressed in years) were more likely to be classified in the cognitive impairment group, with odds ratio 1.189 (95% confidence interval: 1.033–1.368; *p* = 0.016). Females were less likely to exhibit cognitive impairment compared with males, with odds ratio 0.089 (95% confidence interval: 0.010–0.759; *p* = 0.027). Fewer years of education predicted greater likelihood of cognitive impairment, with odds ratio 0.564 (95% confidence interval: 0.384–0.827; *p* = 0.003). Cognitive impairment was more probable in the Southern Planes, with odds ratio 19.334 (95% confidence interval: 2.378–157.181; *p* = 0.006); and in the Southwest region, with odds ratio 37.615 (95% confidence interval: 2.165–653.632; *p* = 0.013), as compared to the Northern Plains.

## Discussion

This study characterized, for the first-time, associations between sleep characteristics and cognitive performance in a sample of aging American Indians. We found that sleep characteristics derived from polysomnography predicted cognitive performance assessed 10 years later. Aspects of sleep were correlated with cognitive performance in nearly all domains investigated by the neuropsychological tests – episodic verbal learning, short-delay and long-delay free recall, processing speed, verbal phonemic fluency, and 3MSE general cognition (Table 4). Furthermore, after adjustment for age, BMI, sex, study site, years of education, CESD depressive symptoms score, and presence of the *APOE e4* allele, longer sleep latency was associated with increased likelihood of cognitive impairment later in life.

Sleep-related disorders have previously been associated with cognitive decline (Suh et al., 2018; Mei et al., 2023), dementia (Robbins et al., 2021a,b), and neurodegeneration (Mullins et al., 2020). Our findings support our initial hypothesis that sleep characteristics are correlated with subsequent cognitive performance in American Indians, such that future cognitive deficits may be predictable from polysomnographically recorded sleep. Longer sleep latency in particular was associated with worse cognitive outcomes, which is consistent with findings in the general population (Suh et al., 2018; Robbins et al., 2021a). Notably, in a nationally representative longitudinal study with 2,812 Medicare beneficiaries in the United States, more than 30 min of sleep latency as well as very short sleep duration (≤5 h) were associated with incident dementia after 5 years (Robbins et al., 2021a). And in a study comprising 2,238 individuals with normal cognition and 655 individuals with mild cognitive impairment in Korea, more than 30 min of sleep latency was associated with increased risk of cognitive decline at 4-year follow-up (Suh et al., 2018). In our study, 23.7% of the sample had more than 30 min of sleep latency. Longer sleep latency was associated with poorer cognitive scores on the COWA test of verbal phonemic fluency – and, importantly, with increased likelihood of cognitive impairment, as defined by more than 1.5 standard deviations below the mean on the 3MSE.

We also found that higher oxygen saturation at baseline was associated with worse total learning, short-delay free recall, and long-delay free recall performance, as measured by the CVLT-SF as well as the WAIS-IV digit symbol coding test at follow-up. These findings were unexpected and inconsistent with what many general population studies have found (Cohen-Zion et al., 2001; Aloia et al., 2003; Spira et al., 2008; Yamout et al., 2012). In our study, surprisingly, associations for all variables related to oxygen saturation and cognitive performance indicated that greater severity of oxygen desaturation was associated with better cognitive performance. We have some hypotheses for these findings. It is possible that participation in the SHHS stimulated awareness of sleep disruptions, particularly in participants meeting criteria for sleep treatment. Thus, those with worse rates of oxygen saturation at baseline may have sought sleep treatment during the approximately 10-year interval between the SHHS and the CDCAI study and, with treatment, improved their cognitive performance. It is also possible that those who had low average oxygen saturation during the SHHS study did not survive to follow up or were too debilitated to participate in the CDCAI study, causing a bias in the results and leaving those who had good average oxygen saturation in the CDCAI study to be relatively more cognitively impaired. However, it is also important to note that the associations between oxygen saturation and cognition in our sample are not statistically robust and may not generalize to other samples. Moreover, since the literature on sleep and cognition in indigenous population is scarce, we used a discovery-based approach with a non-conservative correction for multiple comparisons (FDR), and therefore, we need to consider potential type 1 error. Future studies with larger samples of American Indians that are focused on cognitive disruptions related to sleep are needed to clarify this.

Our study found an influence of study site on associations of sleep with cognitive impairment, even when considering differences in time interval among participants. This raises questions regarding geographical location and its environmental impact or the possibility of inconsistency among sites. All sites included in the study for both the SHHS and CDCAI studies underwent the same training and certification to administer the cognitive tests and perform home polysomnography. The quality of polysomnograms was monitored through central scoring and quality assurance procedures, with no differences observed among study sites (Redline et al., 1998). Nevertheless, future studies should ensure methodological rigidity and consistency of the collected measurements to investigate whether the geographic region of different tribes and communities may have a substantive impact on the associations between sleep quality and cognitive performance.

Our findings highlight the importance of considering subjective and objective measures of sleep in research on sleep and cognition. Different ethnic and racial populations may subjectively experience sleep quality in different ways. In the present study, quantitative measures using polysomnography were better at capturing cognitive performance 10 years later in this population. Given that many determinants of sleep health are modifiable and that sleep is an important population-wide health indicator, public interventions in sleep health can have an impact on mitigating health disparities (Hale et al., 2020). Sleep disorders may be a modifiable risk factor for physical and mental health concerns later in life, including dementia (van Straten et al., 2018; Blackman et al., 2021). As a population facing significant health disparities (Hutchinson and Shin, 2014; Manson and Buchwald, 2021), American Indians have been shown to be at risk for vascular disease (Suchy-Dicey et al., 2017) and cognitive decline (de Souza-Talarico et al., 2016; Cholerton et al., 2019), but studies investigating sleep in this population have been lacking. The current study helps to fill this gap. Documenting sleep characteristics in aging American Indians and determining their relationship with cognitive outcomes provides direction for testing more specific hypotheses in this population, such as whether there is a predictive relationship between mid-life sleep latency and subsequent neurodegeneration, whether sleep treatments would be beneficial for long-term cognitive performance, and whether any deleterious effects of racial and ethnic sleep health disparities are involved.

### Indigenous and racial disparities in sleep

There is a dearth of studies investigating whether sleep patterns in indigenous populations are different than those in other populations. A study involving 31,724 participants (7% Native Hawaiian and Pacific Inlanders, 14% Black individuals, and 79% White individuals) reported that suboptimal sleep duration (less than 6 h per day) occurred more frequently in native Hawaiian and Pacific Inlanders compared to White individuals and Black individuals and was linked to hypertension and diabetes, even after controlling for other variables (Matthews et al., 2018). In our study 41.7% of participants slept less than 6 h per day, and 74.8% slept less than 7 h per day, which is a substantially greater proportion than what has been reported in non-indigenous populations (Kocevska et al., 2021; Sabia et al., 2021). Our sample had a mean sleep duration of 6.19 h (with a SD of 1.23). A meta-analysis with the general population showed that mean sleep efficiency for those 41–65 and > 65 years of age ranged from 88 to 90%, depending on the age group (Kocevska et al., 2021), while in our study, mean sleep efficiency was 77.4% (Table 2). Differences in sample characteristics prevent us from comparing directly between studies. Nonetheless, our data encourage further effort to understand sleep disparities in American Indians. Although there is scarce literature on sleep in American Indians, a systematic review among Indigenous populations in high-income countries indicated that there is a higher incidence of obstructive sleep apnea, and that sleep apnea severity is greater in indigenous compared to non-indigenous populations. A higher prevalence of obesity, medical comorbidity, and health disparities appears to underlie this difference (Woods et al., 2015). A recent study including American Indians, Alaska Natives, Hispanics, and non-Hispanic White older adults with ADRD in Washington State highlighted that difficulty accessing healthcare may explain some of the health disparities (Amiri et al., 2024). The relationship between sleep disorders, cognition and race is complex and likely involves multiple genetic, behavioral and environmental factors. The relationship between sleep and the aging process of older adults from native communities is still unknown. Nonetheless, it is likely that interventions to improve sleep would be beneficial irrespective of race and ethnicity (Egan et al., 2017).

### Limitations

Limitations of the study include a modest sample size and potential bias in our sample. Specifically, the mean 3MSE score for our sample was higher than the mean for the entire CDCAI cohort (Mascarenhas Fonseca et al., 2023), suggesting that there may have been some selection bias, with underrepresentation of individuals with more pronounced cognitive impairment in our sample. Furthermore, we do not have data on whether participants sought sleep treatment during the interval between studies, or regarding reasons for non-participation in the CDCAI study. As such, sample bias needs to be considered when interpreting our findings.

A restricted number of neuropsychological tests was used in the CDCAI study, and there was no baseline cognitive assessment. Sleep and sleep loss effects on cognition are multi-dimensional (Oonk et al., 2008), and important aspects of cognitive performance may have been missed. Moreover, whereas there is broad consensus on how to measure and describe the quantity of sleep in a standardized and reproduceable way (Terzano et al., 2002; Fiorillo et al., 2019; Malhotra et al., 2021), there is debate about the best variables to use when describing sleep quality and the severity of sleep-disordered breathing (Redline et al., 2000; Tucker et al., 2007; Davis et al., 2011). Thus, our selection of sleep and cognition variables may have influenced the results. The lack of sleep assessments at the time of the neuropsychological assessments limits our understanding of whether any changes in sleep during the interval between the SHHS and CDCAI studies (whether from aging, disease, or sleep treatments) may have influenced our findings. And a generalized interpretation of the results was hampered by a lack of clinical diagnoses of sleep disorders and of dementia or prodromal dementia. Furthermore, the PSG recording montage used in SHHS did not include a nasal pressure sensor, which decreases the sensitivity of detecting hypopneic events. However, the impact thereof is not differential and would bias any findings related to sleep disordered breathing to the null rather than the converse. Further, the relatively small sample size hindered the investigation of specific analyses such as a stratified approach for *APOE e4* carriers or sex.

Importantly, even though we found that sleep characteristics measured in mid-life predicted cognitive performance and cognitive impairment approximately 10 years later, that does not necessarily mean that the sleep characteristics in mid-life were causally involved. There is a bidirectional relationship between sleep and cognition, and there may be a common effect in play that links them here. Other factors not included in this study could be driving the observed associations. There is an extensive body of literature indicating that poor sleep may have a profound impact on brain functioning with advancing age (Lim et al., 2014; Borges et al., 2019), and it is likely that this played a role in the present findings as well, but our study design did not allow us to demonstrate that definitively.

## Conclusion

This is the first study to analyze relationships between sleep and cognitive measures in an aging sample of American Indians. We reported novel findings regarding the associations between sleep characteristics, derived from polysomnography and questionnaires, and cognitive performance assessed approximately 10 years later. We found that polysomnography-derived sleep characteristics, but not self-reported sleep measures, collected in mid-life were associated with deficits in cognitive performance in this population later in life. In particular, longer sleep latency was associated with cognitive impairment approximately 10 years later. If further research indicates that sleep latency may serve as a biomarker of cognitive decline with advanced aging, it could provide a straightforward and important tool for early intervention. As American Indians have increased risk for AD and other age-related disorders (Centers for Disease Control and Prevention, 2006; Hutchinson and Shin, 2014; Suchy-Dicey et al., 2017), understanding how poor sleep affects American Indians health across the lifespan is paramount. Sleep and sleep disorders are possible modifiable risk factors for cognitive impairment and dementia, and therefore, are suitable candidates for interventions aimed at preventing neurodegenerative disease development and progression.

## Data availability statement

The data supporting the findings of this study are the intellectual property of the sovereign tribes and communities from whom they were collected and are not directly shareable without the express permission and consent of those entities. Researchers interested in using the data may follow standard Strong Heart Study procedures, per tribal data use agreements. Further inquiries can be directed to the corresponding author.

## Ethics statement

Ethical review and approval were granted for the Strong Heart Study and its ancillary studies. Written informed consent was obtained from all participants. Study procedures for this specific analysis of deidentified data were approved by the appropriate Institutional Research Boards, Tribal Research Review Boards, or Tribal Councils.

## Author contributions

LF: Writing – review & editing, Writing – original draft, Software, Methodology, Investigation, Formal analysis, Conceptualization. MF: Writing – review & editing. NC: Writing – review & editing. NM: Writing – review & editing. DB: Writing – review & editing. HVD: Writing – review & editing. SQ: Writing – review & editing, Supervision. AS-D: Writing – review & editing, Supervision.
